# Biochar and urea enhance phytoremediation of PAH-Pb co-contaminated karst soil: bacterial and metabolic insights

**DOI:** 10.3389/fmicb.2026.1846232

**Published:** 2026-06-12

**Authors:** Hongyu Jin, Juan Zhou, Jing Hu, Shaoqi Zhou

**Affiliations:** 1 College of Resources and Environmental Engineering, Guizhou University, Guiyang, China; 2 Guizhou Provincial Key Laboratory for Prevention and Control of Emerging Contaminants, Guiyang, China

**Keywords:** biochar, PAHs, Pb-contaminated soils, phytoremediation, soil bacterial community

## Abstract

The remediation of polycyclic aromatic hydrocarbon and lead co-contaminated soils in karst regions remains challenging because of strong soil buffering capacity, complex metal speciation, and the distinct transformation mechanisms of organic pollutants and heavy metals. Although urea and biochar are widely used as soil amendments, their interactive effects on plant-assisted remediation and the associated rhizosphere microbial and metabolic mechanisms remain unclear. In this study, urea and biochar were co-applied in a ryegrass pot experiment to evaluate their synergistic effects on the remediation of PAH and Pb co-contaminated karst soil. The combined treatments significantly enhanced PAH degradation within 80 days, with PBCN and PN achieving the highest degradation efficiencies of 49.5 and 38.4%, respectively. Biochar-plant integration also markedly reduced Pb availability, with PBCN showing the greatest reduction, reaching 24.52 mg kg^−1^, which was 36.8% lower than that of the control. The improvement in Pb remediation was closely associated with changes in soil pH, aggregate distribution, and organic matter fractions. The combined application of biochar, urea, and ryegrass increased dehydrogenase, urease, and phosphatase activities, as well as the abundances of 16S rDNA and functional genes. Urea reduced bacterial α-diversity, whereas bacterial community composition was mainly shaped by urea and ryegrass treatments. Network analysis indicated that biochar and urea decreased bacterial network complexity and stability, while ryegrass enhanced rhizosphere network connectivity. Metabolomic analysis further revealed that urea strongly regulated rhizosphere metabolites, particularly organic acids, amino acids, and low-molecular-weight compounds associated with Pb chelation and mobilization. Overall, this study provides new insights into the synergistic remediation of PAH and Pb co-contaminated karst soil by biochar, urea, and ryegrass, and supports the development of green and cost-effective remediation strategies for contaminated karst soils.

## 1 Introduction

Polycyclic aromatic hydrocarbons (PAHs) are ubiquitous organic pollutants primarily derived from anthropogenic activities such as industrial production, pesticide application, waste incineration, and vehicle emissions. Due to their persistence, toxicity, and strong bioaccumulation potential, PAHs have attracted increasing concern regarding their long-term risks to ecosystems and human health (Hofman et al., 2014). Soils act as major sinks for PAHs through atmospheric deposition processes, where their strong adsorption to soil particles leads to prolonged environmental residence and limited natural attenuation.

Soil contamination by lead (Pb), a toxic heavy metal with persistent, non-biodegradable, and highly toxic properties, represents a widespread environmental concern worldwide (Liu et al., 2022). In karst regions, this problem is further exacerbated by the unique soil properties derived from carbonate parent materials, such as high calcium carbonate (CaCO_3_) content, alkaline pH, and strong buffering capacity, which significantly influence Pb speciation, mobility, and bioavailability. Various remediation methods have been developed to mitigate the ecological risks associated with Pb contamination, among which phytoremediation has gained increasing attention as an environmentally sustainable and low-impact strategy (Yan et al., 2020). Notably, phytoremediation has been recognized as one of the ten most promising technologies for improving public health in developing regions (Daar et al., 2002; Dai et al., 2022). Plant species suitable for remediation typically exhibit rapid growth, high biomass production, extensive root systems, and strong tolerance to heavy metal stress. Ryegrass (*Lolium perenne* L.) is considered a representative phytoremediation species and has been widely used in the remediation of heavy metal-contaminated soils due to its high biomass and stress tolerance (Duan et al., 2022; Jiang et al., 2022). In karst soils, Pb is often present in carbonate-bound and residual fractions, which limits its phytoavailability. Therefore, the removal and stabilization of Pb in the rhizosphere are strongly dependent on plant-microbe interactions and rhizosphere processes that regulate Pb transformation and mobilization (Hou et al., 2015).

Biochar has emerged as a multifunctional soil amendment capable of improving soil physicochemical properties and regulating microbial activity (Ameloot et al., 2015; Zhang et al., 2021). Its high surface area and abundant functional groups enable strong sorption, ion exchange, and complexation with heavy metals, thereby reducing Pb mobility and environmental risk (Jin et al., 2018; Wang and Wang, 2019). In addition, biochar can influence rhizosphere conditions, including pH, dissolved organic carbon, and nutrient availability, which may indirectly affect both metal bioavailability and organic pollutant degradation (Jones et al., 2012; Xiao et al., 2018). Despite these advantages, the role of biochar in regulating the coupled remediation of PAHs and Pb in karst soils remains poorly defined.

Water and nutrient management strategies are commonly employed to improve phytoremediation performance (Li et al., 2020a,b). Although urea does not directly interact with Pb, it can significantly influence plant growth and microbial activity by supplying nitrogen. In karst soils, nitrogen availability is often limited due to rapid leaching and low organic matter content, which constrains plant growth and microbial processes. Urea amendment can alleviate nitrogen limitation, promote plant biomass accumulation, and stimulate rhizosphere microbial activity, thereby indirectly affecting Pb transformation and uptake. In addition, nitrogen input may regulate the secretion of root exudates such as organic acids, which can enhance Pb mobilization from carbonate-bound fractions in calcareous soils. Despite these recognized benefits, the combined influence of biochar amendment and nitrogen supplementation on the phytoremediation of Pb-contaminated karst soils remains insufficiently understood. Therefore, elucidating the impacts of biochar-urea co-treatment on plant physiological responses and rhizosphere processes under Pb stress is essential for optimizing phytoremediation strategies.

Soil microorganisms are ubiquitous and play fundamental roles in nutrient cycling, soil structure maintenance, and plant development (Liu et al., 2020). In karst ecosystems, microbial activity is particularly sensitive to changes in soil pH, calcium content, and nutrient availability (Chen et al., 2018). Organic pollutants and heavy metal contamination can alter the structure and function of soil microbial communities, thereby influencing phytoremediation efficiency (Wu et al., 2017). Previous studies have shown that phytoremediation can modify rhizosphere microbial community composition and stimulate the proliferation of metal-resistant microorganisms, which contribute to organic pollutants decomposition, Pb immobilization, transformation, and plant uptake. These findings highlight the critical role of plant-microbe interactions in Pb stabilization and mobilization in karst soils. However, the responses of soil bacterial composition and functional characteristics to combined biochar amendment and nitrogen supplementation during phytoremediation of Pb-contaminated karst soils remain poorly understood.

Therefore, the objectives of this study were to: (1) evaluate the effectiveness of the combined application of biochar, urea, and ryegrass in the remediation of PAHs and Pb co-contaminated karst soil; (2) elucidate the relationships between rhizosphere bacterial community composition, diversity, and their functional roles in Pb transformation and stabilization; and (3) clarify the mechanisms responsible for the enhanced remediation of PAHs under the integrated biochar-nitrogen-ryegrass phytoremediation system.

## 2 Materials and methods

### 2.1 Pot experiment setup

The yellow soil was collected from a karst area (surface layer 0–20 cm) near a lead-zinc mining site in the periphery of Guiyang City. After removing debris such as fallen leaves and twigs, the soil samples were air-dried and passed through a 2 mm sieve for later use. The background lead (Pb) concentration in the soil of this region is approximately 102.19 mg kg^−1^. The preparation of PAH-simulated contaminated soil was primarily conducted by thoroughly mixing the soil with an acetone solution containing phenanthrene and pyrene. After the natural volatilization of acetone, the mixture was aged in the dark for 30 days to obtain simulated contaminated soil with phenanthrene and pyrene concentrations of approximately 30 mg kg^−1^ each.

Eight treatments were established Supplementary Table 1: control (untreated soil), BC (1% biochar), N (1% urea), P (ryegrass only), BCN (1% biochar + 1% urea), PBC (1% biochar + ryegrass), PN (1% urea + ryegrass), and PBCN (1% biochar + urea + ryegrass). The biochar employed in this research was produced from corn straw by pyrolysis at 550 °C under oxygen-limited conditions, and its properties included a pH of 8.3, ash content of 6.8%, water content of 1.04%, organic carbon of 455 g kg^−1^, pyrolysis temperature of 550 °C, total nitrogen of 8.57 mg g^−1^, total phosphorus of 2.24 mg g^−1^, and total potassium of 16.13 mg g^−1^. Analytical-grade urea (99.8% purity) was obtained from BioMaterials Technology Co., Ltd. (Qingdao, China). UUrea and biochar were thoroughly mixed with the soil at the designated ratios and pre-incubated in the dark for at least 7 days prior to planting. Ryegrass (*Lolium perenne* L.) seeds were acquired from Yuanlin Group Co., Ltd., based in Weifang, China. Prior to germination, the seeds were disinfected on the surface using 1% (v/v) sodium hypochlorite and subsequently washed with distilled water. After germination, following a brief pre-cultivation in Hoagland nutrient solution, twelve seedlings of similar size were transferred into plastic pots filled with 500 g of treated soil. Soil water content was maintained at 65% of soil moisture retention at field capacity by regular addition of distilled water. Pots were incubated in a controlled growth chamber under a 10 h light / 14 h dark photoperiod. The experiment lasted 80 days. Each treatment included twelve replicates. At harvest, soil from each pot was divided into two subsamples: one portion was stored at −20 °C for organismal analyses, and the remaining portion was air-dried for physicochemical characterization.

### 2.2 Soil physicochemical property analysis

Soil total PAHs concentrations were determined following previously reported methods (Varjani et al., 2021) with minor modifications. Briefly, soil samples were freeze-dried and extracted with n-hexane for 10 h using a Soxhlet apparatus. The extracts were subsequently concentrated with an evaporator and purified through a column, followed by elution with n-hexane. The eluate was further concentrated and adjusted to a final volume of 1 mL with n-hexane. PAHs were quantified using gas chromatography (GC-MS-QP2020; Shimadzu, Shanghai, China) based on peak area integration. PAHs standards were obtained from Saikerui Technology Co., Ltd. based in Suzhou, China.

Edaphic physicochemical properties were assessed following previously established protocols (Liu et al., 2021). The specific soil physical properties, such as particle size and aggregate distribution; chemical properties, including dissolved organic matter (DOM), pH, electrical conductivity (EC), total nitrogen (TN), total carbon (TC), total sulfur (TS), total hydrogen (TH), total phosphorus (TP), exchangeable nitrate (NO$\begin{array}{c} -\\ 3 \end{array}$-N), and ammonium (NH$\begin{array}{c} +\\ 4 \end{array}$); and biological properties, such as MBC (soil microbial biomass carbon) and MBN (soil microbial biomass nitrogen), were analyzed as described in Supplementary Data Sheet 1.

### 2.3 Assessment of soil enzymatic activities and the abundances of functional genes

Edaphic enzymatic activity was determined following established protocols. The activity of dehydrogenase (DHA) was assessed according to the protocol described by Friedel et al. (1994). Catalase activity (CAT) was assayed using the protocol described by Johansson and Borg (1988), and urease activity was quantified following Tabatabai and Bremner (1972). Lipase activity was determined according to the procedure described by Margesin et al. (2002).

Total DNA from soil samples was isolated immediately after collection by a commercial Soil DNA Kit (Tiangen, China). Quantitative PCR (qPCR) was performed to determine the abundances of bacterial 16S rRNA and fungal ITS genes using primer pairs bacterial-F/bacterial-R and fungal-F/fungal-R, respectively. The qPCR reactions were carried out on an RTQ-960 Pro instrument (ACON, Hangzhou, China).

### 2.4 Soil bacterial community analysis

The V3-V4 hypervariable region of the bacterial 16S rRNA gene was amplified using universal primers 341F and 785R. PCR amplifications were performed on an HM Gene Amp^®^ PCR System (Hengmei, China).

Raw sequencing reads were demultiplexed and quality-filtered using QIIME (Caporaso et al., 2010) according to the following criteria: (1) reads were truncated to achieve an average quality score ≥20, with sequences shorter than 50 bp removed; (2) reads containing ambiguous bases were discarded; and (3) only sequences with alignments longer than 10 bp relative to the reference were retained, while unaligned reads were excluded.

Rarefaction analyses were performed using MOTHUR, and Venn diagrams were generated in R to visualize shared and unique OTUs among treatments. OTU clustering and soil bacterial community richness and diversity indices. Taxonomic assignment of OTUs was conducted against the Greengenes database, and dominant phyla and genera were identified to characterize the composition of the soil bacterial communities.

### 2.5 Plant trait analysis

Plant samples were collected at the conclusion of the 80-day pot experiment. Shoot and root lengths were measured using a ruler, and plant dry biomass was determined after drying at 105 °C for 24 h. Methods for rhizosphere soil metabolomic analysis are detailed in the Supplementary Data Sheet 2.

For chlorophyll and carotenoid quantification, 0.2 g of fresh, clean leaves were cut into approximately 5 mm × 1 mm filaments and placed in glass tubes wrapped with black plastic to prevent light exposure. Ten milliliters of absolute ethanol were added dropwise, and the tubes were incubated in the dark at room temperature for approximately 3 days, until the leaf tissues became colorless. Absorbance values were then measured using a spectrophotometer (EU-2600D, ONLAB, Shanghai, China) at 664, 646, and 471 nm to determine concentrations of chlorophyll a, chlorophyll b, and carotenoids, respectively.

### 2.6 Statistical analysis

Once the assumptions of variance homogeneity and data normality were verified, differences among treatments in soil enzyme activities, functional gene abundances, and bacterialα-diversity indices were evaluated using one-way analysis of variance (ANOVA). Variations in soil physicochemical properties across treatments were analyzed using three-way ANOVA using Data Processing System (DPS) software, version 8.90.

Unless otherwise specified, subsequent analyses are conducted in R software. To evaluate bacterialβ-diversity, we employed principal coordinate analysis (PCoA) on the OTU dataset; Principal component analysis (PCA) was used to interpret soil metabolic profiles.

Bacterial co-occurrence networks were constructed to explore potential associations among taxa. To ensure data robustness, only taxa present in more than two-thirds of the samples per treatment were retained. Pairwise Spearman correlation coefficients were then calculated, and only correlations with an absolute coefficient (|*r*|) ≥ 0.8 and a *p-value* < 0.05 were retained for network construction (Harrell and Harrell, 2019). Networks were constructed and visualized using Python (v. 3.9). Network robustness was evaluated by computing natural connectivity under sequential node removal, also performed in Python (v. 3.9) (Python Software Foundation, United States).

## 3 Results

### 3.1 Effect of remediation treatments on soil PAHs and lead

**Figure 1:**
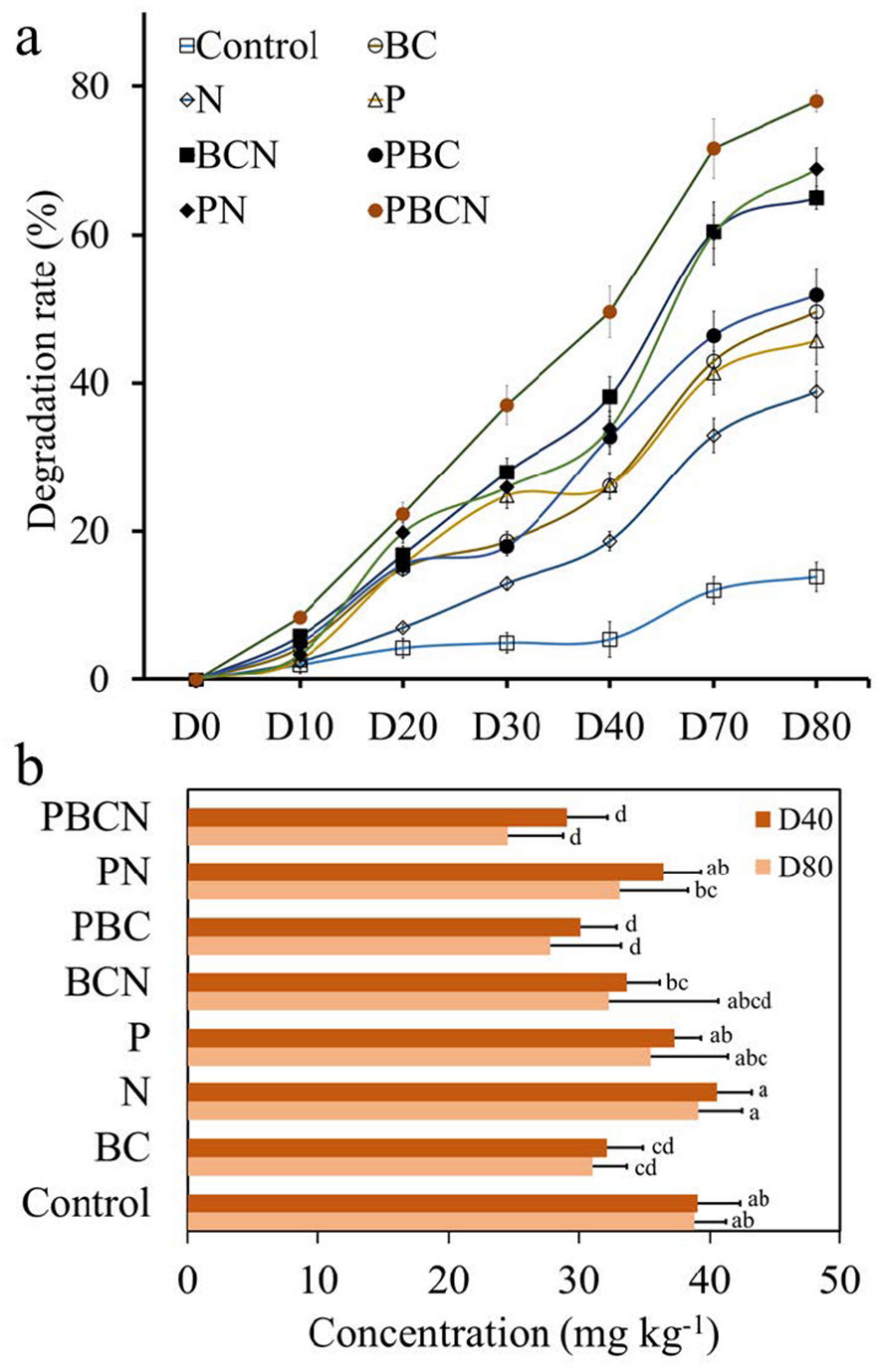
**(a)** Temporal variations in the soil total PAHs degradation rate from day 10 to day 80 during the incubation period. **(b)** Available Pb concentration in soil on day 40 and 80.

Residual PAH concentrations and bioavailable lead levels varied considerably among the different treatment groups. Figure 1a presents the temporal dynamics of PAH degradation rates in soil samples throughout the 80-day incubation period. All combined treatment regimens demonstrated a progressive enhancement in degradation rates over the initial 60-day period. Among these, the PBCN and PN treatments exhibited the highest degradation rates, surpassing the sole treatment with biochar (BC) and urea (N), which reached 49.5 and 38.4%, respectively. After 80 days, all treatments resulted in significantly lower PAH concentrations compared to the control.

Figure 1b summarizes the concentrations of available Pb in soil under different treatments at day 40 (D40) and day 80 (D80). Relative to the control (38.82 mg kg^−1^ at D80), all amended treatments except N alone reduced Pb availability. The BC treatment lowered the available Pb concentration to 31.05 mg kg^−1^ by D80. Biochar (BC) significantly immobilized Pb, reducing its available concentration to 31.05 mg/kg by D80. The integration of plants and biochar (PBC) further enhanced this effect (27.84 mg kg^−1^). Notably, the PBCN treatment achieved the highest remediation efficiency, with available Pb dropping to a minimum of 24.52 mg kg^−1^ at D80, a 36.8% decrease relative to the control. Overall, available Pb levels in all plant-involved treatments followed a clear temporal downward trend from D40 to D80, suggesting a synergistic effect between biochar immobilization, nitrogen-stimulated growth, and phytoremediation.

### 3.2 Soil physicochemical properties

Different remediation treatments exerted distinct effects on soil physicochemical properties. Supplementary Figure 1 display the distribution patterns of soil particle sizes ranging from 0 to 1.2 mm. The control treatment exhibited the greatest mean particle diameter, followed sequentially by the BC, P, N, and PBCN treatments. In untreated soil, the proportions of aggregates within the size ranges of 1.0–2.0 mm, 0.25–1.0 mm, and below 250 μm accounted for 49.3, 30.8, and 8.9%, respectively. Under the PBCN amendment, the corresponding values shifted to 38.4, 33.6, and 14.0% Supplementary Figure 1.

**Figure 2:**
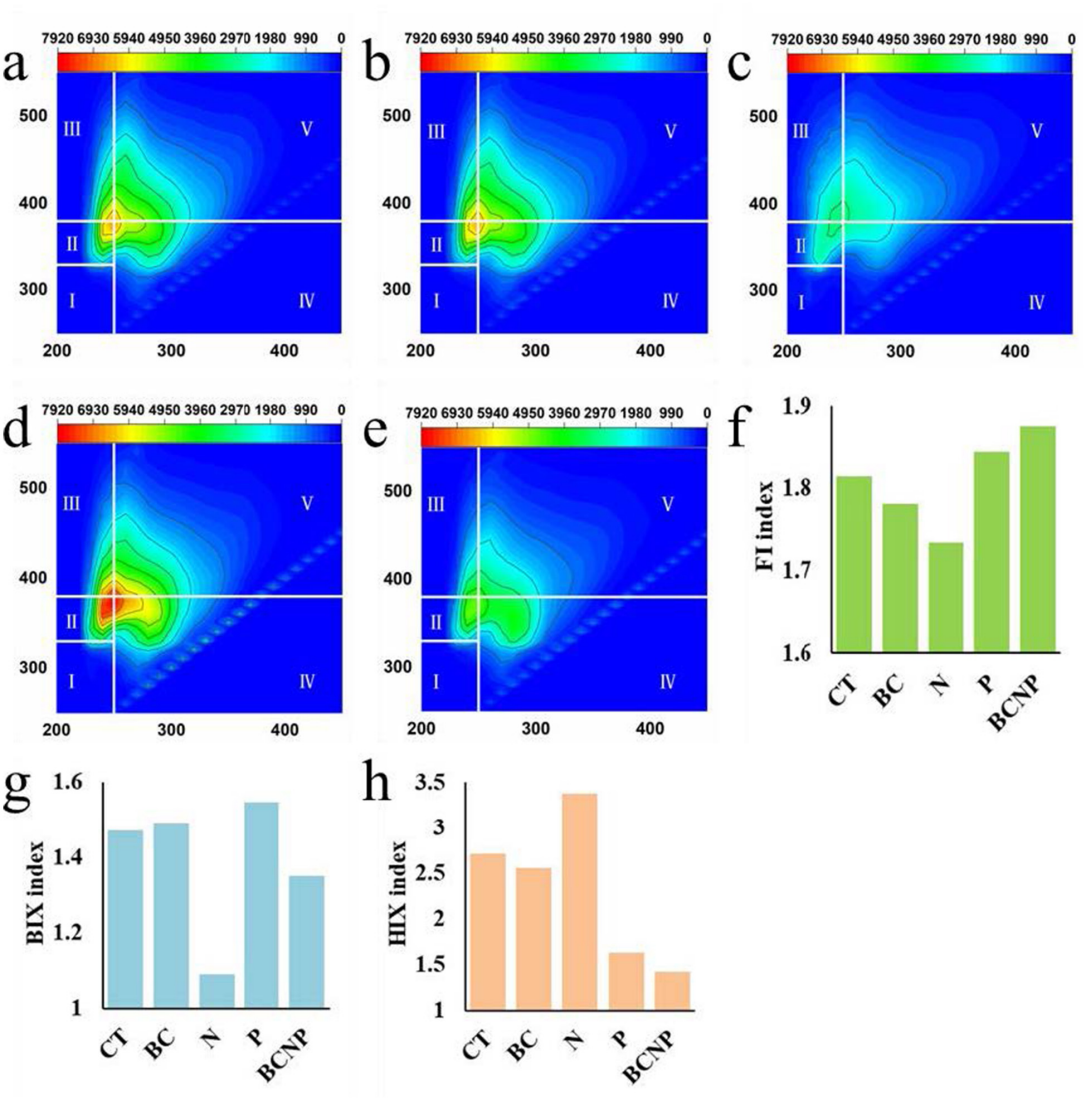
Three-dimensional excitation-emission matrix (3D-EEM) spectra of dissolved organic matter (DOM) isolated from soils subjected to CT **(a)**, BC **(b)**, N **(c)**, P **(d)**, and PBCN **(e)** treatments. Treatment-wise comparisons of the fluorescence index **(f)**, biological index **(g)**, and humification index **(h)**.

Figures 2a–h details the molecular signatures of dissolved organic matter via 3D-EEM fluorescence and associated optical parameters. Compared to the control group, the PBCN intervention led to a substantial decline in the fluorescence response across multiple regions (II, III, IV, and V). This treatment also altered the DOM source characteristics, as evidenced by a diminished HIX and BIX alongside a concurrent elevation in the FI values. These divergent index trends suggest a transformation in the humification state and biological availability of the organic pool following PBCN application.

The alterations in soil physicochemical profiles under various experimental conditions are detailed in Table 1. The findings indicate that biochar incorporation was associated with changes in soil microbial biomass carbon (MBC), total phosphorus (TP), sulfur (TS), total carbon (TC), and pH. Meanwhile, the introduction of urea exerted multifaceted impacts (*p* < 0.05) on a broad spectrum of parameters, notably nitrogenous compounds (both NH$\begin{array}{c} +\\ 4 \end{array}$ and NO$\begin{array}{c} -\\ 3 \end{array}$), electrical conductivity (EC), and elemental compositions (TN, TC, TS, TH, TP). Similarly, ryegrass cultivation was shown to significantly shift the soil pH and EC, while also reshaping the total hydrogen and nitrogen pools and the microbial biomass phosphorus (MBP). The combined application of biochar, urea, and ryegrass was associated with concurrent changes in ammonium, total nitrogen, phosphorus, pH, and electrical conductivity.

**Table 1:** Soil physicochemical properties under the different groups.

Treatment	pH	EC (μS cm^−1^)	TC (g kg^−1^)	TN (g kg^−1^)	TS (g kg^−1^)	TH (g kg^−1^)	TP (g kg^−1^)	NH$\begin{array}{c} +\\ 4 \end{array}$ (mg kg^−1^)	NO$\begin{array}{c} -\\ 3 \end{array}$(mg kg^−1^)	MBC (mg kg^−1^)	MBN (mg kg^−1^)	MBP (mg kg^−1^)
Control	8.02 ± 0.02^d^	1366 ± 25^a^	19.5 ± 2.1^c^	0.44 ± 0.03^f^	0.54 ± 0.04^bcd^	0.18 ± 0.05^c^	0.61 ± 0.01^a^	1.85 ± 0.4^d^	12.7 ± 2.9^a^	95.5 ± 2.3^a^	25.5 ± 1.6^a^	3.0 ± 0.1^a^
BC	8.01 ± 0.01^d^	1382 ± 13^a^	24.4 ± 0.9^ab^	0.56 ± 0.02^e^	0.50 ± 0.02^cd^	0.87 ± 0.20^bc^	0.58 ± 0.01^a^	1.01 ± 0.1^d^	13.5 ± 2.5^a^	103.3 ± 1.4^a^	25.8 ± 0.2^a^	2.9 ± 0.1^a^
N	8.74 ± 0.04^a^	1296 ± 39^b^	20.1 ± 0.6^c^	0.80 ± 0.02^c^	0.48 ± 0.06^d^	1.76 ± 1.0^b^	0.61 ± 0.04^a^	30.3 ± 7.2^a^	0.35 ± 0.04^c^	101.1 ± 3.4^a^	27.6 ± 1.4^a^	3.3 ± 0.6^a^
P	8.21 ± 0.02^b^	1184 ± 18^c^	23.5 ± 0.6^b^	0.69 ± 0.02^d^	0.77 ± 0.03^a^	5.49 ± 0.05^a^	0.51 ± 0.01^b^	8.4 ± 0.4^c^	1.04 ± 0.02^c^	97.1 ± 2.2^a^	27.3 ± 3.5^a^	3.0 ± 0.2^a^
BCN	8.71 ± 0.03^a^	1385 ± 24^a^	25.6 ± 0.3^a^	0.90 ± 0.04^b^	0.45 ± 0.1^d^	1.72 ± 1.2^b^	0.60 ± 0.02^a^	29.4 ± 2.6^a^	0.48 ± 0.09^c^	107.7 ± 3.5^a^	26.8 ± 1.2^a^	3.1 ± 0.1^a^
PBC	8.24 ± 0.02^b^	1185 ± 29^c^	23.5 ± 0.4^b^	0.64 ± 0.01^d^	0.58 ± 0.02^bc^	4.68 ± 0.11^a^	0.61 ± 0.01^a^	16.3 ± 0.5^b^	1.37 ± 0.03^c^	102.2 ± 15.9^a^	29.9 ± 0.8^a^	3.9 ± 0.9^a^
PN	8.12 ± 0.02^c^	1379 ± 21^a^	23.4 ± 0.2^b^	1.09 ± 0.02^a^	0.60 ± 0.02^b^	5.64 ± 0.36^a^	0.60 ± 0.01^a^	19.0 ± 0.4^b^	2.86 ± 0.04^bc^	104.8 ± 8.2^a^	26.8 ± 4.3^a^	3.4 ± 0.8^a^
PBCN	8.24 ± 0.02^b^	1308 ± 11^b^	24.5 ± 0.3^ab^	0.84 ± 0.02^c^	0.46 ± 0.02^d^	4.83 ± 0.1^a^	0.61 ± 0.01^a^	13.9 ± 0.4^b^	4.44 ± 0.06^b^	108.6 ± 9.8^a^	27.0 ± 2.4^a^	4.0 ± 0.4^a^

Values are presented as mean ± standard deviation (SD) from three independent replicates (*n* = 3). Statistical analyses were performed using one-way analysis of variance (ANOVA) followed by Waller-Duncan *post-hoc* test. For data that did not meet the assumption of normality, the Kruskal–Wallis test was applied. Different superscript lowercase letters (a, b, c, d, e, f) within the same column indicate significant differences among treatment groups at *p* < 0.05. The same letter denotes no statistically significant difference.

### 3.3 Soil enzyme activities and functional gene abundances

**Figure 3:**
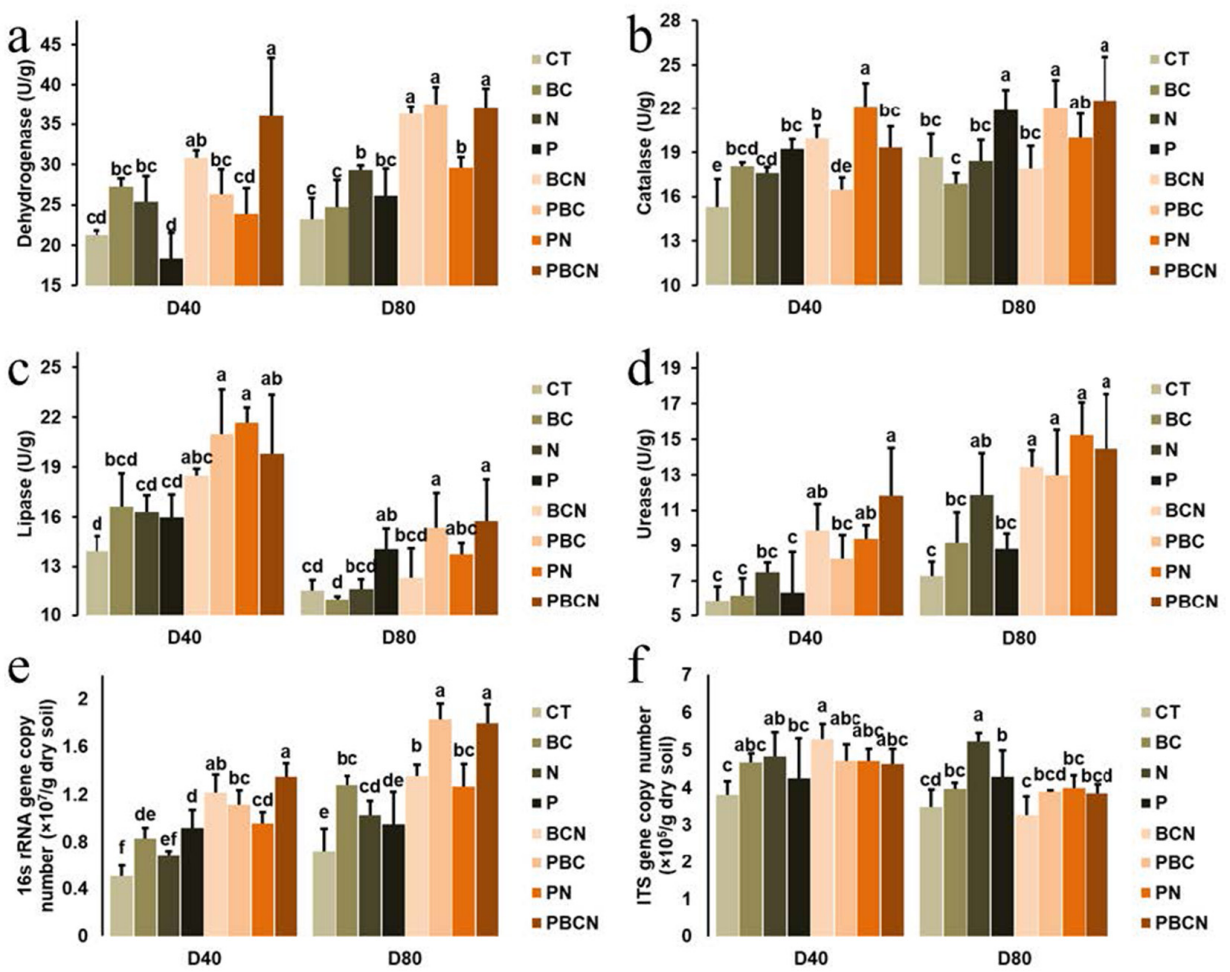
Temporal variations in soil enzyme activities and functional gene abundances at days 40 and 80 under different remediation treatments. **(a)** Dehydrogenase activity; **(b)** catalase activity; **(c)** lipase activity; **(d)** urease activity; **(e)** 16S rRNA gene abundance; **(f)** ITS gene abundance. Error bars indicate standard deviations (*n* = 3). Columns annotated with different letters differ significantly among treatments (one-way ANOVA, *p* < 0.05).

Soil enzymes play an essential role as catalysts, indicating the decomposition of pollutants by soil organisms. The dehydrogenase activity (DHA) in soil (Figure 3a) was higher at day 80 compared to day 40 across all treatments. At day 40, the DHA activity was significantly higher in the BCN and PBCN treatments than control. At day 80, DHA activity was significantly higher in remediation treatments relative to the control.

For catalase activity (CAT) (Figure 3b), significant increases were observed at day 40 in remediation groups compared to the control. At day 80, the CAT activity was enhanced in remediation groups compared with CT (*p* < 0.05).

Lipase activity (Figure 3c) showed a decline at day 80 compared to day 40. At day 40, compared to the control, lipase activity was increased in the BCN, PBC, PN, and PBCN treatments (*p* < 0.05). At day 80, lipase activity was higher in P, PBC, and PBCN treatments compared to the control.

Soil urease activity (Figure 3d) was higher at day 80. At initial, urease activity was elevated in the BCN, PN, and PBCN treatments compared to CT. At day 80, urease activity was significantly higher in remediation treatments compared to the control.

Quantitative PCR (qPCR) analysis of functional genes was performed to evaluate the abundance of key microorganisms involved in pollutant degradation. The abundance of the 16S rRNA gene was significantly higher in the BC, BCN, PBC, PN, and PBCN treatments than in the control group at both time points (Figure 3e). For the ITS gene (Figure 3f), significant increases were observed at day 40 in the BCN treatment compared to the control. At last, fungal gene abundance was higher in urea and ryegrass treatments compared with the CT group (*p* < 0.05).

### 3.4 Soil bacterial community

Soil bacterial communities were analyzed after sequencing, and the Shannon index revealed that bacterial diversity was significantly lower in the N, BCN, PN, and PBCN treatments compared to the control Supplementary Figure 2; *p* < 0.05. Principal coordinate analysis (PCoA) indicated that axes 1 and 2 together accounted for 76.5% of the total variation in bacterial community composition among the treatments.

Soil bacterial profiles revealed that Gammaproteobacteria, Actinobacteria, Bacilli, and Alphaproteobacteria constituted the primary bacterial classes regardless of the treatment applied. At the genus level, the most prevalent taxa identified were *Pseudomonas, Lysobacter, Lactobacillus, Alcanivorax, Luteimonas, Sporosarcina*.

**Figure 4:**
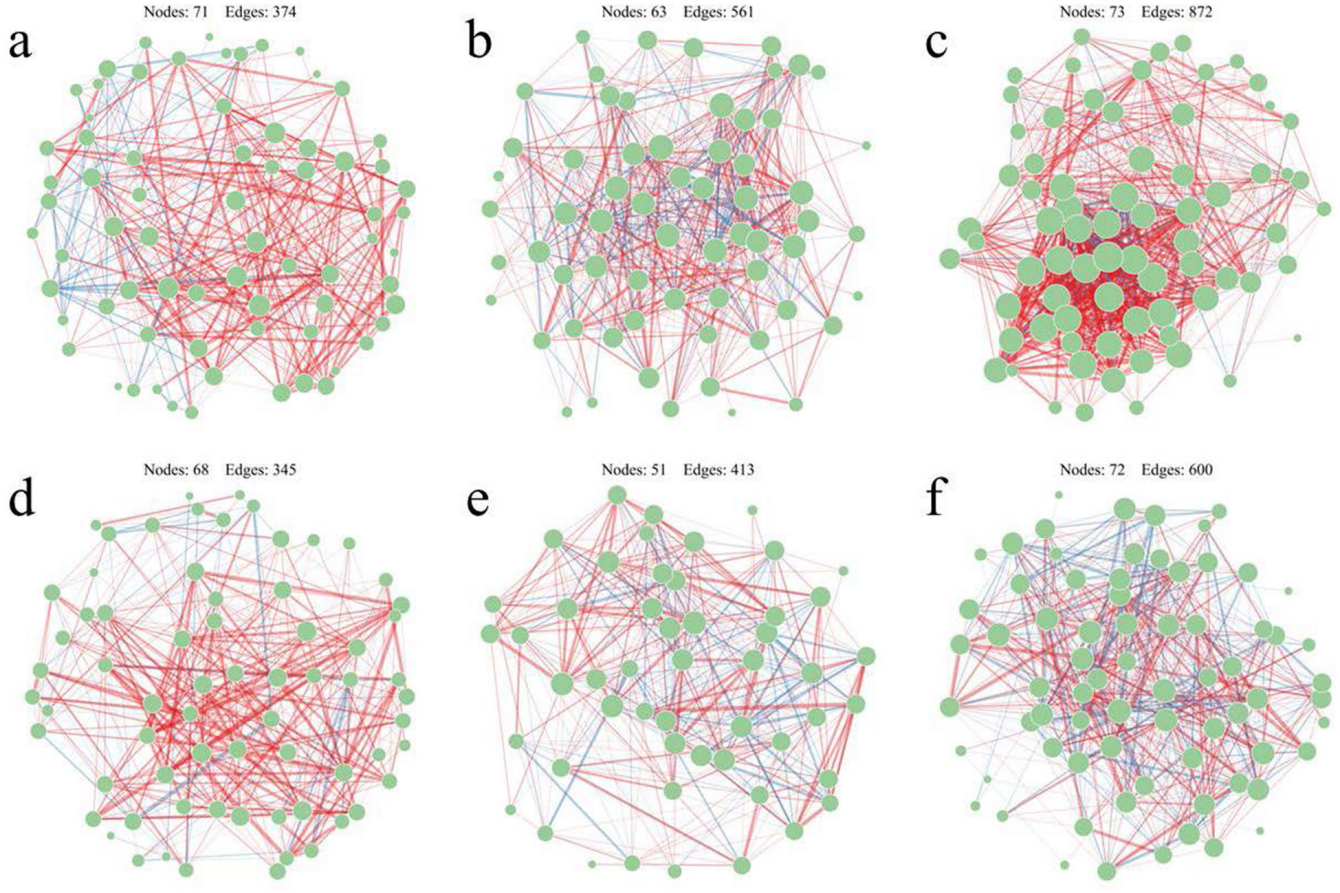
Co-occurrence network visualization of soil bacterial communities undertreatments without biochar **(a)**, urea **(b)**, ryegrass **(c)** and with the application of biochar **(d)**, urea **(e)**, or ryegrass **(f)**. Each node represents an individual taxon, and edges denote significant Spearman correlations between taxa; red and blue connections indicate positive and negative associations, respectively.

**Figure 5:**
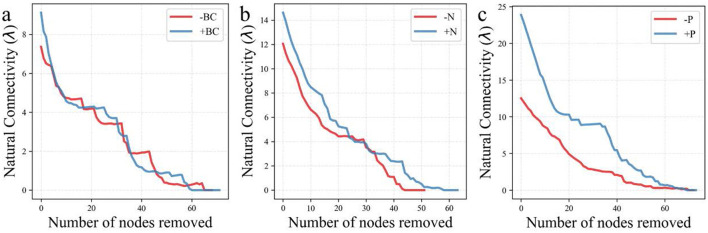
Bacterial network stability following the addition of biochar **(a)**, urea **(b)**, and ryegrass **(c)**. + and − represent addition or not.

Bacterial co-occurrence networks were constructed using Spearman correlation coefficients to explore potential associations among taxa. Based on the updated network analysis, the addition of biochar was associated with a decrease in the number of nodes and edges. Specifically, the network without biochar contained 71 nodes and 374 edges, whereas the biochar-amended network contained 68 nodes and 345 edges (Supplementary Table 2). Similarly, urea addition was associated with changes in network structure. The network without urea comprised 63 nodes and 561 edges, while the urea-amended network contained 51 nodes and 413 edges. The proportion of positive correlations increased in the presence of urea, indicating a potential shift toward more positive associations. In contrast, ryegrass cultivation was associated with an increase in network size. The network without plants (Figure 4c) had 73 nodes and 872 edges, whereas the network with ryegrass (Figure 4f) contained 72 nodes and 600 edges. Notably, the number of edges decreased in the presence of ryegrass, suggesting altered association patterns among bacterial taxa. Additionally, the results shown in Figure 5 indicate that urea addition and ryegrass cultivation enhanced the robustness of the soil microbial network, whereas biochar showed no significant effect.

### 3.5 Plant properties and rhizosphere soil metabolome

The results of the pot experiment indicated that the addition of biochar or urea significantly promoted ryegrass growth Supplementary Figure 3. Shoot length was highest in the PBC and PBCN treatments, while root length and plant dry biomass were greater in the PBC, PN, and PBCN treatments compared to the control (*p* < 0.05). Analysis of plant biochemical traits showed that leaf chlorophyll a and b contents were higher in the PN and PBCN treatments than in the control (*p* < 0.05), and carotenoid content was elevated in the PBC, PN, and PBCN treatments relative to the control (*p* < 0.05).

**Figure 6:**
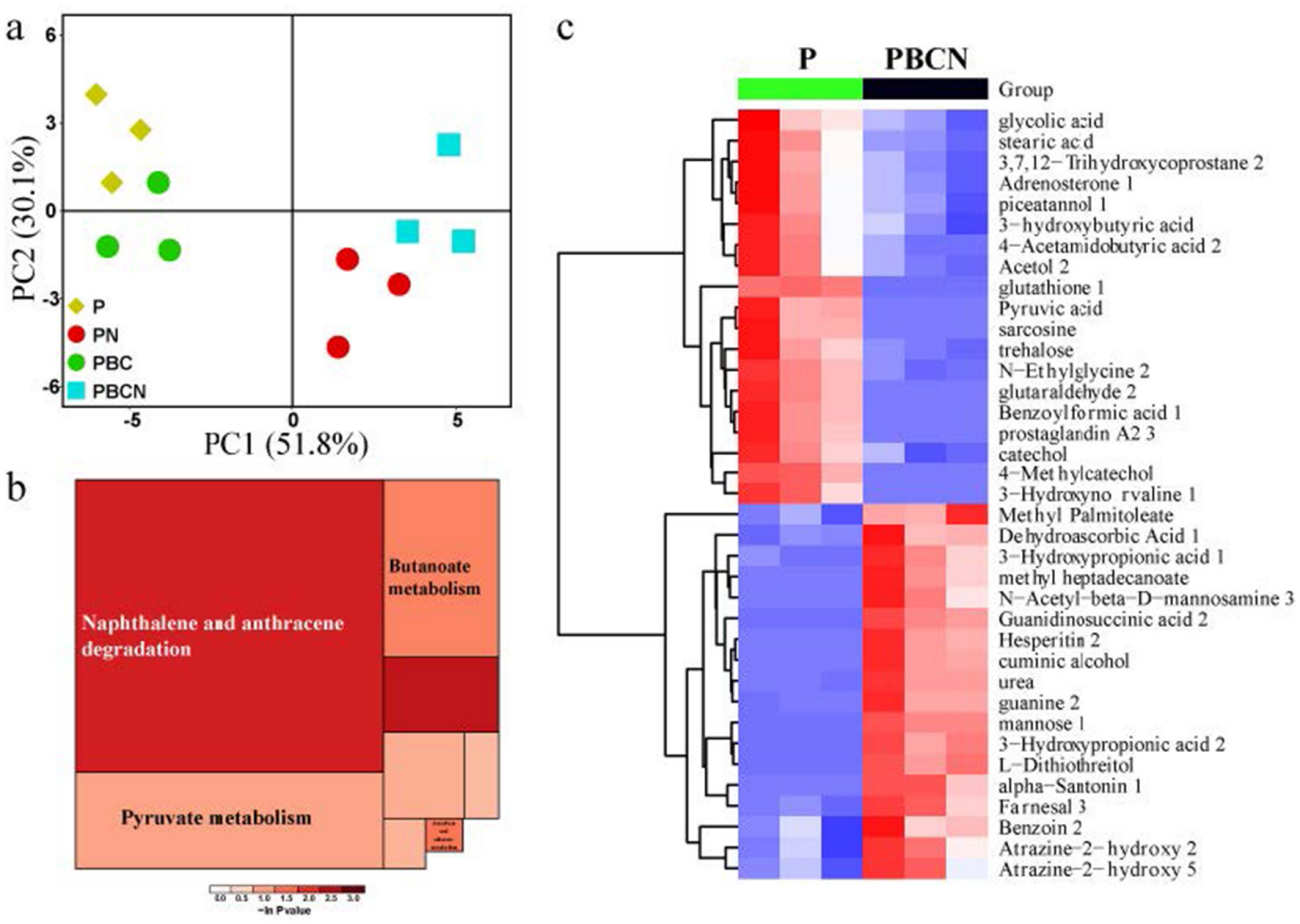
Metabolomic profiling of rhizosphere soils subjected to the P, PN, PBC, and PBCN treatments. **(a)** Principal component analysis (PCA) illustrating the overall variation in metabolite composition among treatments. **(b)** Heatmap depicting differentially abundant metabolites between the P and PBCN groups. **(c)** Treemap summarizing the metabolic pathways associated with differential metabolites in rhizosphere soil under the P vs. PBCN treatments.

Figure 6 illustrates the metabolomic profiles of rhizosphere soils under the P, PN, PBC, and PBCN treatments. Principal component analysis (PCA) indicated clear separation of metabolite compositions among the four treatments (Figure 6a). Compared with biochar, urea exerted a stronger influence on the rhizosphere soil metabolome. The heatmap (Figure 6b) shows that the P treatment was enriched with 19 metabolites, such as glutathione, pyruvic acid, and sarcosine, while the PBCN group was enriched with 18 metabolites, including urea, methyl hexadecanoate, and benzoin. The treemap (Figure 6c) highlights the distinct metabolic pathways in the rhizosphere soil between the P and PBCN treatments, showing upregulation of butanoate, pyruvate metabolism, anthracene and naphthalene degradation metabolism in PBCN treatment.

## 4 Discussion

### 4.1 Effects of remediation agents on soil PAHs removal, Pb stabilization, and physicochemical properties

Bioremediation, which can transform organic contaminants such as PAHs into CO_2_ and H_2_O, is considered a sustainable strategy. In this study, the PBCN treatment resulted in the highest PAH removal and the greatest reduction in soil available Pb. However, the mechanisms underlying these two processes are fundamentally different and are therefore discussed separately. For PAH degradation, the process is primarily driven by microbial activity. The observed increase in dehydrogenase and lipase activities (Figures 3a, c) and the enrichment of putative hydrocarbon-degrading taxa such as Lysobacter in the PBCN treatment suggest enhanced microbial catabolic potential. The shift from macro-aggregates to micro-aggregates Supplementary Figure 1 may have increased the surface area available for microbial colonization and improved contact between microorganisms and PAHs, thereby facilitating biodegradation.

For Pb, the reduction in available Pb concentration is likely governed by physicochemical processes rather than direct biological transformation. The alkaline pH of biochar (8.3) and the high CaCO_3_ content of the karst soil may promote Pb precipitation as carbonates or hydroxides. Additionally, the functional groups on biochar surfaces can complex Pb^2+^ ions, reducing their bioavailability. The presence of ryegrass may also influence Pb speciation through root exudates or changes in rhizosphere pH, but the primary mechanism is likely immobilization via sorption and precipitation rather than uptake or transformation.

In this study, PBCN treatment significantly removed PAHs and decreased the content of soil available Pb. Both BC and PBCN treatments shifted soil structure from macro-aggregates to micro-aggregates (Awad et al., 2018), likely enhancing contaminant exposure. EEM spectra further indicated a depletion of various DOM components and a lower humification index in the PBCN treatment, reflecting a more labile organic matter pool (Ohno, 2002). These findings suggest that PBCN acts as a catalyst to prime bacterial activity and overcome kinetic barriers, thereby facilitating the mineralization of PAHs. A limitation of this study is the use of a single amendment dosage, which prevents evaluation of dose-response relationships and may limit the generalizability of the results.

Soil physicochemical properties inevitably shifted during the remediation process, and these changes were generally consistent with contaminant dissipation trends. The optimal C/N/P ratio is widely recognized to be approximately 100:10:1 (US Environmental Protection Agency, 2002). After 80 days, the control soil exhibited C/N and C/P ratios of 43:1 and 32:1, respectively, whereas the PBCN treatment adjusted these ratios to 28:1 and 39:1, indicating conditions that may be more favorable for microbial activity. Effective phytoremediation systems typically require soils with suitable physicochemical and biological conditions that can support plant growth and microbial activity, which may contribute to enhanced contaminant attenuation (Hajabbasi, 2016).

### 4.2 Responses of soil enzyme activities, bacterial community and ecological interactions to remediation agents

Extracellular enzymes are involved in the oxidative transformation of recalcitrant organic compounds (Rong et al., 2021). In this study, biochar amendment and ryegrass cultivation were associated with increased dehydrogenase and lipase activities, which coincided with higher PAH removal (Figure 3). However, enzyme activity measurements provide indirect evidence and do not confirm specific degradation pathways. Future studies employing enzyme inhibition assays or metatranscriptomic analyses would help establish causal links. However, it should be noted that enzyme activity measurements provide indirect evidence and cannot be used to confirm specific degradation pathways or mechanisms. Further in-depth molecular-level investigations are still required, such as enzyme isolation or functional assays in controlled systems, to better elucidate the underlying biochemical processes.

Soil bacterial communities are fundamental to bioremediation processes (Wu et al., 2020). Nitrogen addition significantly reduced bacterial α-diversity in this study Supplementary Figure 2, consistent with meta-analyses reporting that urea fertilization can decrease microbial diversity (Geisseler and Scow, 2014). Such discrepancies may reflect differences in soil type and remediation context. A decrease in alpha diversity does not necessarily indicate reduced functional potential, as nitrogen addition may selectively enrich microbial taxa involved in contaminant degradation. Notably, the genus *Lysobacter* in the PBCN treatment. This taxon is known to produce biosurfactants that increase the bioavailability of hydrophobic hydrocarbons and facilitate their degradation (Hayward et al., 2010). Network analysis further showed that nitrogen addition simplified bacterial network structure and reduced robustness (Figures 4, 5), although it increased the proportion of positive interactions, suggesting a shift toward more cooperative microbial associations (Li B. B. et al., 2021). While sophisticated analytical frameworks were employed to explore the links between bacterial community dynamics and PAH degradation, these correlations do not constitute conclusive evidence. Future efforts will focus on the isolation and cultivation of functional hydrocarbon-degrading bacteria to experimentally validate the hypotheses derived from our data analysis. It should be noted that this study focused on bacterial communities based on 16S rRNA sequencing, while fungal and archaeal communities were not investigated, which may also contribute to PAHs degradation and nitrogen cycling.

### 4.3 Plant growth promotion and rhizosphere effects

Rhizodegradation is considered an important process in the remediation of organically contaminated soils, which is associated with enhanced contaminant availability and root system development. Biochar is also widely recognized as an environmentally beneficial soil amendment (Sohi et al., 2010; Zhang et al., 2019). In this study, both biochar and urea significantly promoted ryegrass growth, as indicated by increased shoot and root length, biomass, and chlorophyll content Supplementary Figure 3. Improved plant performance may have been associated with enhanced rhizosphere activity, which could in turn contribute to the higher PAH removal observed under integrated treatments.

Despite these observations, this study has certain limitations. In particular, root system development was not quantitatively characterized using root scanning or imaging techniques. Given that root architecture may influence rhizosphere processes, this represents an important aspect for future investigation.

Root exudation and the release of labile carbon from biochar may increase soil dissolved organic carbon pools (Zhao et al., 2019), which in turn can influence microbial community composition. Conversely, changes in microbial communities may also be associated with shifts in soil metabolite profiles, suggesting potential coupling between microbiota and metabolome (Hewavitharana et al., 2021).

### 4.4 Bacterial-metabolite interactions and study limitations

Metabolomic analysis in this study indicated that urea had a relatively stronger effect on rhizosphere metabolite composition compared with biochar. In addition, pathways related to aromatic hydrocarbon degradation, such as naphthalene and anthracene degradation, as well as pyruvate metabolism, were enriched in the combined treatments. These changes may reflect altered microbial metabolic activity potentially associated with enhanced transformation of organic pollutants (Chen et al., 2022). It should be noted that the interpretations in this study are primarily based on correlation analyses and do not establish causal relationships. Further experimental validation is required to confirm the underlying mechanisms. This study was conducted under short-term controlled pot conditions; therefore, extrapolation to long-term field-scale remediation should be made with caution.

## 5 Conclusions

The integrated amendment of biomass-derived char, nitrogen fertilizer, and *Lolium perenne* achieved the greatest degradation efficiency of PAHs among all treatments. Relative to the control, combined treatments reduced the mean soil particle size and altered the composition of DOM. Concurrently, the activities of hydrocarbon-degrading enzymes and the abundances of functional genes associated with pollutant degradation were markedly enhanced under the integrated application. Although urea addition decreased soil bacterial α-diversity, the overall profile structure was primarily shaped by combined influence of urea-N and ryegrass. In addition, rhizosphere metabolomic profiles were strongly modified by nitrogen and biochar inputs, and the co-application of urea and biochar promoted the activation of metabolic pathways involved in organic pollutant transformation. Collectively, these results provide an effective strategy for enhancing phytoremediation of PAHs and Pb co-contaminated soils and elucidate the physicochemical-bacterial-metabolic mechanisms underlying the synergistic effects of biochar and nitrogen amendments.

## Data Availability

The datasets presented in this study can be found in online repositories. The names of the repository/repositories and accession number(s) can be found in the article/Supplementary material.
