# Supplementary material for: Biochar and urea enhance phytoremediation of PAH-Pb co-contaminated karst soil: bacterial and metabolic insights
Source: Front Microbiol. 2026 Jun 12;17:1846232. doi: 10.3389/fmicb.2026.1846232 (PMC13303467; doi:10.3389/fmicb.2026.1846232)
Supplement: Supplementary file 1 [file Table_1.DOCX]

**SUPPLEMENTARY MATERIALS**

**BIOCHAR AND UREA ENHANCE PHYTOREMEDIATION OF PAH-PB CO-CONTAMINATED KARST SOIL: BACTERIAL AND METABOLIC INSIGHTS**

Hongyu Jin ^a,b^, Juan Zhou ^a,b^, Jing Hu ^a,b^, Shaoqi Zhou ^a,b,*^

^a^ *College of Resources and Environmental Engineering, Guizhou University, Guiyang 550025, China*

^b^ *Guizhou Provincial Key Laboratory for Prevention and Control of Emerging Contaminants, Guiyang 550025, China*

^*^Corresponding author. E-mail: [zhousq210607@163.com](mailto:zhousq210607@163.com) (S.Z.)

X1 Method for soil physicochemical properties analysis

Soil particle size distribution was measured using a laser particle size analyzer (NS-90Z, Saiman, China), while soil aggregate distribution was determined by wet sieving using a rotary sieving device. Dissolved organic matter (DOM) was characterized via excitation-emission matrix (EEM) fluorescence spectroscopy (Fluo Imager, LDI, Estonia) with a xenon lamp as the light source at room temperature (25 ± 2 °C). Soil pH was measured at a 1:5 (w/v) soil-to-water ratio using a pH meter (PHE-2C, Shanghai, China), and electrical conductivity (EC) was determined with a conductivity meter (DDSJ-319L, China). Total carbon (TC), total nitrogen (TN), total hydrogen (TH), and total sulfur (TS) were quantified using an elemental analyzer (Flash Smart, Italy). Total phosphorus (TP) was measured using the molybdenum blue method following digestion with sulfuric and perchloric acid solutions. Exchangeable ammonium (NH4+) and nitrate (NO3--N) were extracted with 1 mol L-1 potassium chloride solution and analyzed using a CFA 1100 continuous flow analyzer (Atta Lab, Changshu, China). Soil microbial biomass carbon (MBC) and nitrogen (MBN) were determined via the chloroform fumigation-extraction method.

X2 Method for metabolomic analysis of rhizosphere soil

*Metabolite Extraction*

A 1000 ± 5 mg aliquot of sample was weighed into a 5 mL centrifuge tube. Subsequently, 1 mL of pre-chilled extraction solvent (methanol/dH₂O, 3:1, v/v) and 1 mL ethyl acetate (EA) containing 5 μL of internal standard solution (2-chloro-L-phenylalanine, 1 mg mL⁻¹) were added. The mixture was vortex-mixed for 30 s, homogenized using a ball mill at 40 Hz for 4 min, and subjected to ultrasonication in an ice-water bath for 5 min; this sonication step was repeated three times. After centrifugation at 4 °C for 15 min at 10 000 rpm (≈9600 × g; rotor radius 8.6 cm), the supernatant was transferred into 5 mL microcentrifuge tubes.

The residue was re-extracted by adding another 1 mL of the pre-cooled methanol/dH₂O (3:1, v/v) solution and 1 mL EA, followed by repeating the vortexing, homogenization, ultrasonication, and centrifugation procedures described above. All supernatants were pooled, and a 2 mL aliquot was transferred to a clean tube. For preparation of the quality control (QC) sample, 600 μL from each extract was combined to obtain a representative pooled sample.

Following solvent removal in a vacuum concentrator, the dried residues were subjected to methoximation by adding 30 μL methoxyamine hydrochloride solution (20 mg mL⁻¹ in pyridine) and incubating at 80 °C for 30 min. Samples were then derivatized with 40 μL BSTFA reagent (1% TMCS, v/v) at 70 °C for 1.5 h. After cooling to room temperature, 5 μL of fatty acid methyl ester (FAME) solution in chloroform was added to the QC sample. Finally, all derivatized extracts were analyzed using gas chromatography-time-of-flight mass spectrometry (GC-TOF-MS).

*GC-TOF-MS Analysis*

GC-TOF-MS measurements were conducted on an Agilent 7890 gas chromatography system interfaced with a time-of-flight mass spectrometer and equipped with a DB-5MS fused-silica capillary column. A 1 μL aliquot of each derivatized extract was introduced in splitless injection mode. High-purity helium served as the carrier gas, with a front inlet purge flow of 3 mL min⁻¹ and a constant column flow of 1 mL min⁻¹. The oven temperature program was initially held at 50 °C for 1 min, ramped to 310 °C at 10 °C min⁻¹, and maintained at 310 °C for 8 min. The injector and transfer line temperatures were both set at 280 °C, whereas the ion source temperature was maintained at 250 °C. Electron impact ionization was operated at −70 eV. Mass spectral data were acquired in full-scan mode over an m/z range of 50-500 at an acquisition rate of 12.5 spectra s⁻¹, following a solvent delay of 6.25 min.

*Data preprocessing and annotation*

Raw chromatographic data were processed using ChromaTOF software (v4.3x, LECO) for peak detection, baseline correction, deconvolution, retention time alignment, and peak integration. Metabolite annotation was achieved by comparing both mass spectra and retention indices against the LECO-Fiehn Rtx5 reference library. To ensure data reliability, features detected in fewer than 50% of quality control (QC) samples or exhibiting a relative standard deviation (RSD) greater than 30% in QC samples were excluded from subsequent analyses.

Fig. S1. Soil aggregate size distribution (0-1200 μm) under the CT, BC, N, P, and PBCN treatments (a).
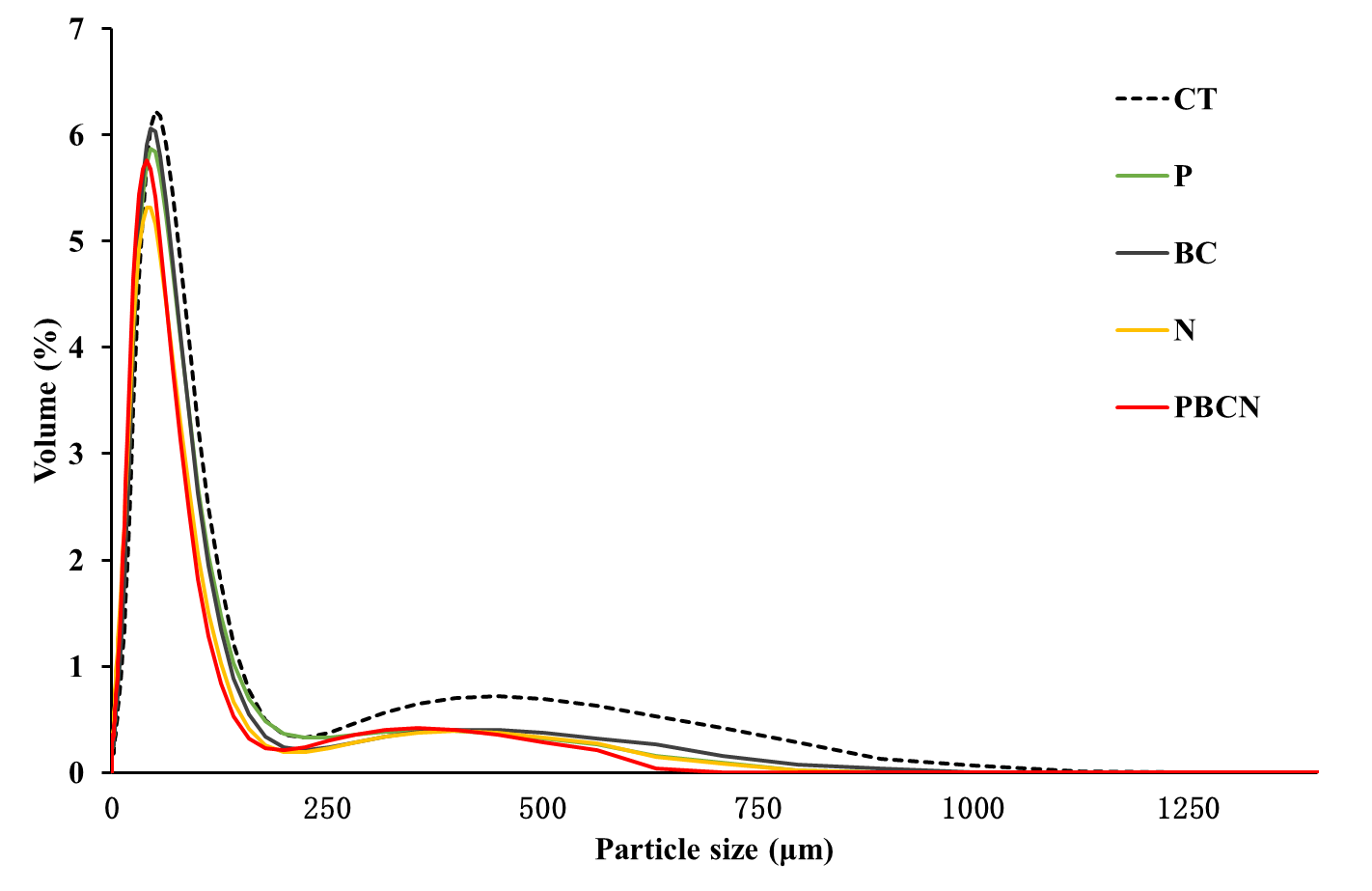
Fig. S2. (a) Soil bacterial Shannon diversity index; distinct letters above the boxplots denote statistically significant differences among treatments. (b) Principal coordinates analysis (PCoA) plot illustrating variations in soil bacterial β-diversity. (c) Chord diagram representing the relative abundance of soil bacterial communities at the class level. (d) Stacked bar chart depicting the relative abundance of soil bacterial taxa at the genus level.
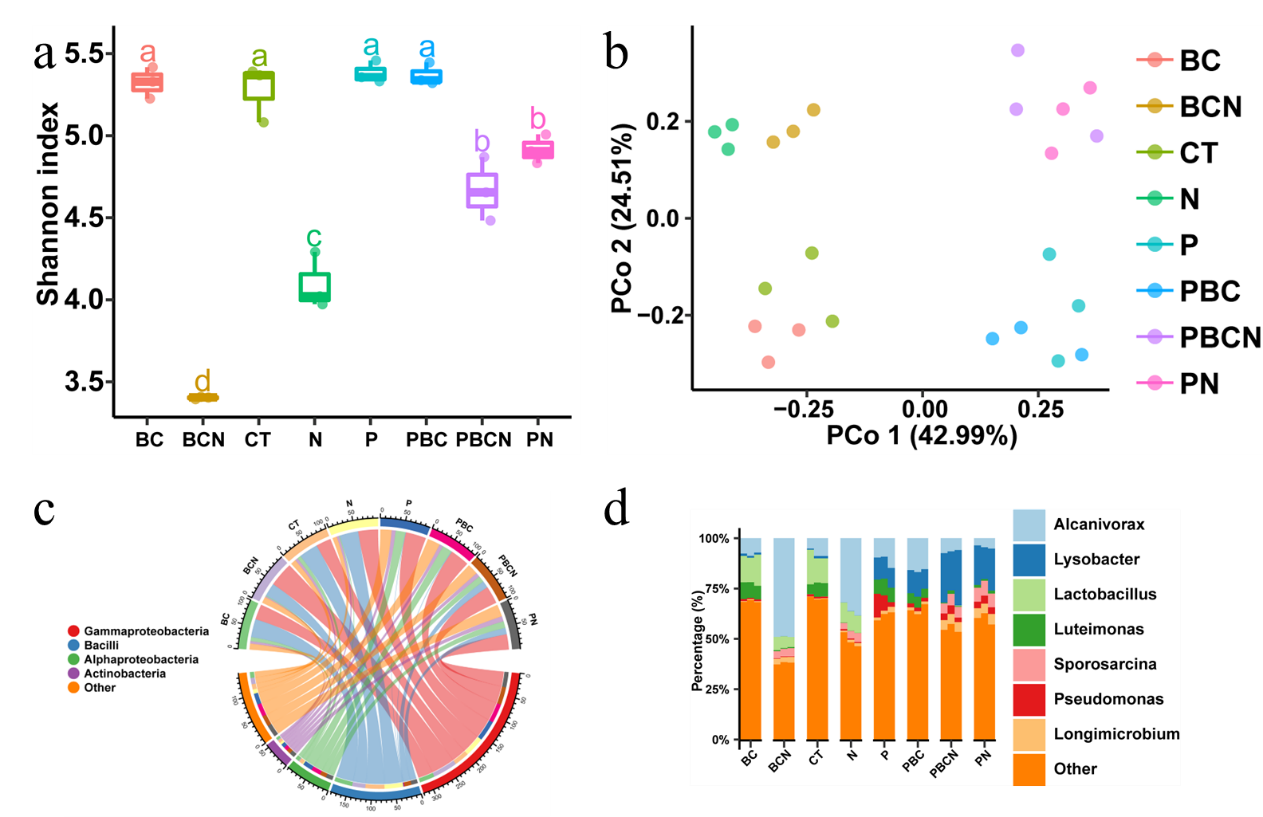


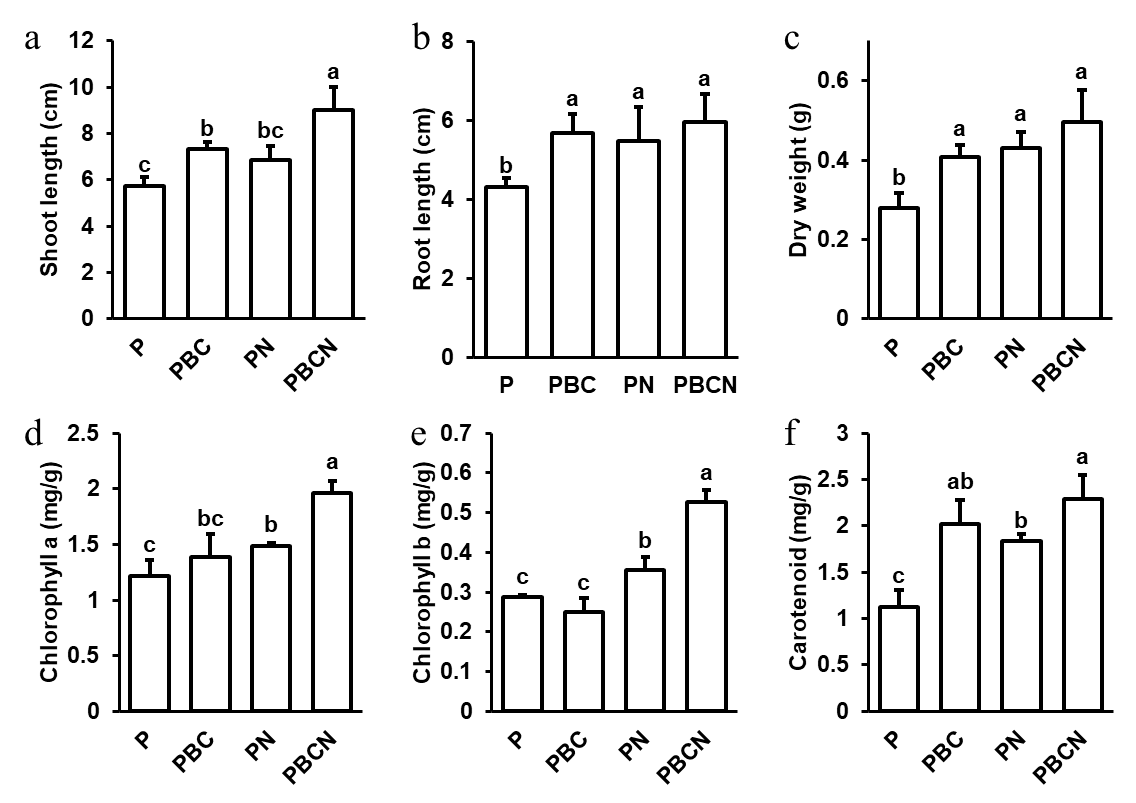
Fig. S3. Growth performance and photosynthetic pigment characteristics of ryegrass under different remediation treatments. (a) shoot length; (b) root length; and (c) shoot dry biomass of ryegrass seedlings. (d) Chlorophyll a, (e) Chlorophyll b, and (f) Carotenoid contents in ryegrass leaves across the four treatments.

Table S1. Experimental treatment design; “U” and “N” indicate utilization and non-utilization, respectively.

| Treatments | Biochar | Urea | Ryegrass |
| --- | --- | --- | --- |
| Control | N | N | N |
| BC | U | N | N |
| N | N | U | N |
| P | N | N | U |
| BCN | U | U | N |
| PBC | U | N | U |
| PN | N | U | U |
| PBCN | U | U | U |

| Networks | Nodes | Links (+%) | Network density | Average degree | Average clustering coefficient | Average path length | Network diameter | Modularity |
| --- | --- | --- | --- | --- | --- | --- | --- | --- |
| -BC | 71 | 374 (80.74%) | 0.150503 | 10.53521 | 0.669545 | 3.05674 | 8 | 0.557261 |
| +BC | 68 | 345 (84.05%) | 0.151449 | 10.14706 | 0.645086 | 3.194231 | 7 | 0.631818 |
| -N | 63 | 561 (63.45%) | 0.28725 | 17.80952 | 0.577404 | 1.90681 | 5 | 0.304003 |
| +N | 51 | 413 (61.50%) | 0.323922 | 16.19608 | 0.590151 | 1.832157 | 4 | 0.319504 |
| -P | 73 | 872 (77.86%) | 0.331811 | 23.89041 | 0.694475 | 1.874429 | 4 | 0.334553 |
| +P | 72 | 600 (54.5%) | 0.234742 | 16.66667 | 0.540028 | 2.020704 | 5 | 0.36169 |

Table S2. Topological properties of soil microbial networks.
